# The use of natural language processing for the identification of ageing syndromes including sarcopenia, frailty and falls in electronic healthcare records: a systematic review

**DOI:** 10.1093/ageing/afae135

**Published:** 2024-07-06

**Authors:** Mo Osman, Rachel Cooper, Avan A Sayer, Miles D Witham

**Affiliations:** AGE Research Group, Translational and Clinical Research Institute, Faculty of Medical Sciences, Newcastle University, Newcastle upon Tyne, UK; NIHR Newcastle Biomedical Research Centre, Newcastle upon Tyne NHS Foundation Trust, Cumbria Northumberland Tyne and Wear NHS Foundation Trust and Newcastle University, Newcastle upon Tyne, UK; AGE Research Group, Translational and Clinical Research Institute, Faculty of Medical Sciences, Newcastle University, Newcastle upon Tyne, UK; NIHR Newcastle Biomedical Research Centre, Newcastle upon Tyne NHS Foundation Trust, Cumbria Northumberland Tyne and Wear NHS Foundation Trust and Newcastle University, Newcastle upon Tyne, UK; AGE Research Group, Translational and Clinical Research Institute, Faculty of Medical Sciences, Newcastle University, Newcastle upon Tyne, UK; NIHR Newcastle Biomedical Research Centre, Newcastle upon Tyne NHS Foundation Trust, Cumbria Northumberland Tyne and Wear NHS Foundation Trust and Newcastle University, Newcastle upon Tyne, UK; AGE Research Group, Translational and Clinical Research Institute, Faculty of Medical Sciences, Newcastle University, Newcastle upon Tyne, UK; NIHR Newcastle Biomedical Research Centre, Newcastle upon Tyne NHS Foundation Trust, Cumbria Northumberland Tyne and Wear NHS Foundation Trust and Newcastle University, Newcastle upon Tyne, UK

**Keywords:** ageing syndromes, electronic healthcare records, natural language processing, informatics, systematic review, older people

## Abstract

**Background:**

Recording and coding of ageing syndromes in hospital records is known to be suboptimal. Natural Language Processing algorithms may be useful to identify diagnoses in electronic healthcare records to improve the recording and coding of these ageing syndromes, but the feasibility and diagnostic accuracy of such algorithms are unclear.

**Methods:**

We conducted a systematic review according to a predefined protocol and in line with Preferred Reporting Items for Systematic reviews and Meta-Analyses (PRISMA) guidelines. Searches were run from the inception of each database to the end of September 2023 in PubMed, Medline, Embase, CINAHL, ACM digital library, IEEE Xplore and Scopus. Eligible studies were identified via independent review of search results by two coauthors and data extracted from each study to identify the computational method, source of text, testing strategy and performance metrics. Data were synthesised narratively by ageing syndrome and computational method in line with the Studies Without Meta-analysis guidelines.

**Results:**

From 1030 titles screened, 22 studies were eligible for inclusion. One study focussed on identifying sarcopenia, one frailty, twelve falls, five delirium, five dementia and four incontinence. Sensitivity (57.1%–100%) of algorithms compared with a reference standard was reported in 20 studies, and specificity (84.0%–100%) was reported in only 12 studies. Study design quality was variable with results relevant to diagnostic accuracy not always reported, and few studies undertaking external validation of algorithms.

**Conclusions:**

Current evidence suggests that Natural Language Processing algorithms can identify ageing syndromes in electronic health records. However, algorithms require testing in rigorously designed diagnostic accuracy studies with appropriate metrics reported.

## Key Points

- The recording and coding of ageing syndromes such as sarcopenia, frailty and falls in hospital records is known to be suboptimal.
- It is possible to use Natural Language Processing algorithms to identify ageing syndromes in electronic healthcare records.
- Diagnostic accuracy for ageing syndromes appears to be acceptable based on the available evidence.
- There are, however, important limitations on study design quality and the reporting of accuracy metrics.

## Introduction

Ageing syndromes are a set of clinical conditions that commonly occur in older adults and are of major importance to the health and wellbeing of older people [1]. Ageing syndromes consist of recognisable sets of signs and symptoms [2], such as sarcopenia, frailty, falls, delirium, dementia, incontinence and multiple long-term conditions (MLTCs; also known as multimorbidity) [3]. The identification of people with these syndromes is important in clinical practice to ensure that they are able to access relevant interventions, care and support services that can improve quality of life and chances of remaining independent and reduce risk of premature mortality [4]. Accurate identification and recording of these syndromes is essential not only to deliver high-quality individual care but also to help inform service planning and research for these syndromes [5].

The recording and coding of ageing syndromes in hospital records has been demonstrated to be poor in comparison with some other medical conditions [5]. Previous research has found systematic under-reporting of common ageing syndromes such as falls [6], incontinence and pressure ulcers [7]. Several reasons for this have been posited. For example, nonspecialist staff are likely to underdiagnose ageing syndromes, and even in cases where a diagnosis is made, these may not be accurately captured in the clinical notes according to structured clinical coding standards such as ICD-10 or SNOMED-CT [8–10]. Additionally, the coding systems based on these standards may not be adequate for coding complex comorbidity and syndromes associated with ageing [11]. For example, MLTCs may not be recorded as an entity in notes and do not have a specific ICD-10 code as they are not a single condition, and the component conditions contributing to MLTCs may themselves be inadequately coded.

### Natural Language Processing

Natural Language Processing (NLP) [12] offers a potential solution to improve identification of certain ageing syndromes in electronic healthcare records (EHRs). NLP is a broad term that describes the use of computational, statistical and machine-learning techniques for the automated analysis of human language to derive meaning or generate text [12, 13].

There are numerous methods of NLP that have clinical applications [14]. For example, text classification NLP algorithms aim to classify texts into predefined categories, such as whether or not a given text is associated with a patient who has a particular diagnosis [15]. This classification can be achieved through various approaches. One such example is the application of rule-based algorithms. Here, a set of manually constructed rules classify a text based on predefined features. These features could be the presence or absence of words that are indicative of signs or symptoms associated with a particular disease or condition. The manually constructed rules require specialist input, and their development can be time consuming, particularly for complex classifications.

An alternative method for text classification is through the use of machine learning classification. In this method, a series of steps are applied to represent the vocabulary in a text as a set of features (or predictor variables) that can be used in the training of machine learning classifiers that aim to predict the class or category of a text [16]. The machine learning algorithms learn relationships between feature-sets and classes, and use these learned relationships to predict classifications in unseen data. There are numerous machine learning algorithms suitable for text classification ranging from support vector machines to neural networks.

The performance of text classification algorithms is often assessed by dividing a dataset used for the training of an algorithm into a ‘training’ dataset and a ‘test’ dataset (this is often referred to as a train-test split) [17]. In the ‘test’ dataset, predictions are compared with ‘labels’ that represent the ground truth. In a clinical context, these ‘labels’ may be diagnoses assigned by clinical experts after manual text review.

Another NLP method that has useful clinical applications is Named Entity Recognition (NER). Rather than aiming to classify texts into categories, this method aims to identify occurrences of terms or concepts in clinical texts, such as diagnoses, medications or procedures. NER can therefore be particularly helpful for identifying diagnoses that have been recorded in unstructured clinical texts but have not been captured according to structured clinical coding standards [18].

There have been many studies in the literature that have used NLP methods for the identification of diagnoses in EHR, and whilst the application of these methods has been reviewed for identifying other disease groups such as psychiatric conditions [19–21], the feasibility and diagnostic accuracy of NLP for identifying ageing syndromes are unclear. In addition, there are many different methods of applying NLP and the comparative effectiveness of these has also not been evaluated for ageing syndromes. This review aims to systematically review published studies that have assessed feasibility and diagnostic accuracy of NLP algorithms for identifying ageing syndromes in electronic healthcare records (EHRs).

## Methods

We conducted a systematic review according to a prespecified protocol and according to PRISMA (Preferred Reporting Items for Systematic Reviews and Meta-Analyses) guidelines. The protocol for this systematic review was registered on the PROSPERO (International Prospective Register of Systematic Reviews) database (registration number: CRD42022307069).

### Data sources and search strategy

Systematic searches were conducted in the following databases: PubMed, Embase, CINAHL, ACM digital library, IEEE Xplore and Scopus. The searches were run from the inception of each database to the end of September 2023. No language restrictions were applied to the searches. The search strategy was developed by the first author M.O. and was then peer reviewed by a specialist liaison librarian with experience in both computing and medical literature databases, who checked the search strategy and suggested modifications. The final search strategies for the included databases are provided in Appendix Table S1.

### Article selection

Articles were eligible for inclusion in this review if the mean age of study participants was 18 or over. We included studies that reported feasibility (a description of whether it was possible to successfully apply the NLP algorithms to textual data from an EHR). Additionally, studies were required to report NLP methodology and performance/diagnostic accuracy metrics such as precision [(Positive Predictive Value (PPV)], recall (sensitivity), F1-score, specificity, Negative Predictive Value (NPV) and accuracy. Studies were included if they applied to any of the following ageing syndromes: sarcopenia, frailty, falls, delirium, dementia, incontinence and multimorbidity/MLTCs. Studies were included if they used data belonging to patients in the following contexts: hospital inpatients, secondary care outpatients, primary care patients, community-dwelling individuals and care home residents. No exclusion criteria for specific patient groups were applied. However, we excluded studies that used NLP to predict the future onset of conditions or complications of conditions. Studies where only an abstract was available without a full-text paper (e.g. conference abstracts) were also excluded.

### Screening, extraction and quality assessment

After combining and deduplicating the database searches, all titles were independently screened by two reviewers (M.O. and M.D.W.). If either reviewer believed the title should be included or was uncertain, the abstract of the study was retrieved for further scrutiny. The same process was then applied to abstract screening, with full-text papers retrieved if either reviewer judged the study should be included or was uncertain. In the final stage, each reviewer recorded whether they believed the article met the review’s inclusion criteria and recorded the reasons for exclusion if they felt the article did not meet the inclusion criteria. Differences in assessment at the full-text screening stage were resolved by consensus.

Data extraction was performed for selected articles by reviewer M.O. using a data extraction form as shown in Appendix material S1. Each data extraction form was checked by the second reviewer M.D.W., and any differences in assessment were resolved by consensus.

Quality assessment of all studies was performed using the QUADAS-2 tool, which is recommended for the evaluation of risk of bias and applicability of primary diagnostic accuracy studies [22]. Whilst the aims of this systematic review were broader than simply assessing diagnostic accuracy, this was one of the primary aims, and therefore, QUADAS-2 was selected as the closest appropriate tool available for assessing the quality of studies in this context.

Due to the diversity of ageing syndromes and NLP methods, we decided at the protocol planning stage that meta-analysis would not be appropriate for this review. Therefore, we applied the Synthesis Without Meta-analysis (SWiM) reporting guideline principles [23].

We generated tables to present study population characteristics including the number of participants for each study, sex, age, ethnicity, country of study and healthcare setting. We also generated tables summarising the methods of each study including the ageing syndrome, NLP methodology, source of text and method of validation. Finally, we presented metrics including precision (positive predictive value; PPV), recall (sensitivity), specificity, F1-score and accuracy [24]. These metrics are indicative of the performance of NLP algorithms and their diagnostic accuracy. It was not possible to conduct further analyses on the results due to the heterogeneity of the studies given that they focused on a variety of ageing syndromes and used varying NLP methodologies. We therefore presented the performance metrics for studies grouped by ageing syndrome and also grouped by NLP methodology. NLP methods were categorised according to whether they intended to classify texts or only identify specific words or phrases (Named Entity Recognition) [25]. Among text classification methods, we further categorised the methods according to whether they used a rule-based approach, machine learning or deep learning [26].

## Results

**Figure 1:**
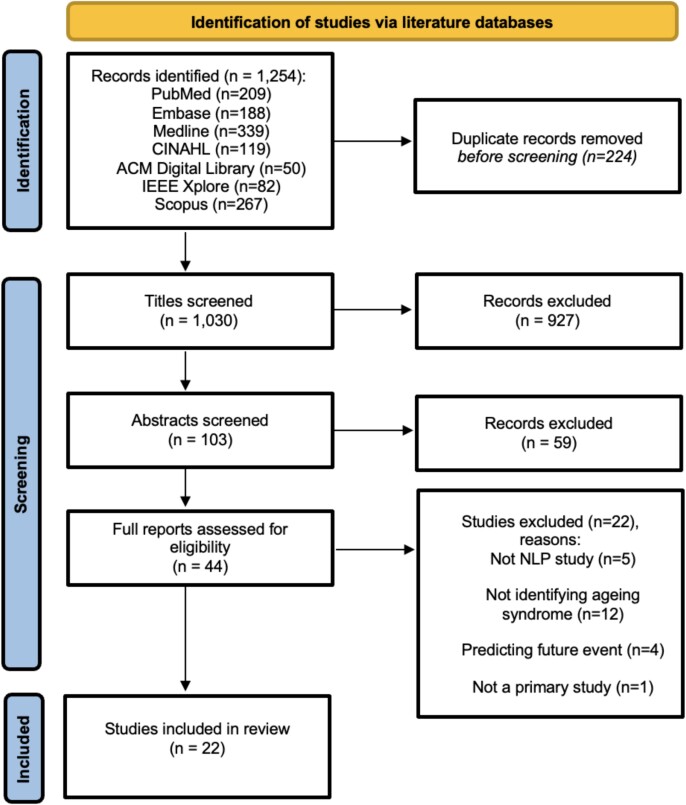
PRISMA 2020 flow diagram for identification and screening of records.

After deduplication, the literature searches identified 1030 records. Nine hundred twenty-seven records were excluded at the title screening stage, and 59 records were excluded at the abstract screening stage, leaving 44 studies for full-text review. Of these, 5 were excluded due to not using NLP methods, 12 were excluded due to not studying one of the ageing syndromes specified in this review, 4 were excluded due to using NLP for predicting future events and 1 was excluded due to being a review article and not a primary study. After full-text screening, there were 22 studies [27–48] selected for inclusion in the systematic review as shown in Figure 1.

The details of these studies including population demographics are shown in Table 1. One study used NLP to identify a composite diagnosis of sarcopenia and/or frailty, 12 to identify falls, 5 to identify delirium, 5 to identify dementia and one to identify incontinence. No studies attempted to identify multimorbidity. These studies used a variety of NLP methods, 19 of which were categorised as text classification, and three studies were categorised as using named entity recognition (NER) methods as shown in Table 2. These studies used a variety of methods for testing and validation of the NLP algorithms that are also specified in Table 2. The majority of studies (14/22) used a train-test split for testing of the NLP algorithms, and only four studies included external validation as part of their testing/validation strategy. Manual text review by clinical experts or trained annotators was the most popular gold-standard comparator and was used in the majority of studies (18/22). The application of NLP algorithms on text documents from EHRs was found to be feasible in all 22 studies. Appendix Table S2 gives more details on the methods used for the NLP algorithms and their testing.

**Table 1:** Study details including population characteristics

Study	*n* participants	Female sex (%)	Mean age (years)^a^	Ethnicity (%)	Healthcare setting	Country of study
Jarman 2010 [27]	247	NR	NR	NR	Hospital outpatients	USA
Toyabe 2012 [28]	NR	NR	NR	NR	Academic hospital	Japan
McCart 2013 [29]	2241	NR	NR	NR	Ambulatory care	USA
Reuben 2017 [30]	989	NR	NR	NR	NR	USA
Kharrazi 2018 [31]	18 341	58.9%	76 (SD 7.5)	NR	Multiple healthcare settings (home, hospital, ED)	USA
Chen 2019 (a) [32]	185	NR	NR	NR	Large multi-specialty medical group	USA
Chen 2019 (b) [33]	185	NR	NR	NR	Large multi-specialty medical group	USA
Gori 2019 [34]	553	0%	62.4 (SD 5.2)	White/Caucasian (75%) Black (3%) Asian/pacific islander (9%) Other/unknown (13%)	NR	Italy/USA
Patterson 2019 [35]	NR	NR	NR	NR	Hospital ED	USA
Topaz 2019 [36]	89 459	NR	NR	NR	Homecare visits	USA
Dolci 2020 [38]	240	NR	69.3 (range: 18–103)	NR	Internal medicine department	Switzerland
Moorthi 2020 [39]	NR	59%	Median 74.9 (IQR: 62.2–84.8)	White (69%), Black (10%), Other (21%)	Multiple healthcare settings (multi-health system database)	USA
Patterson 2020 [40]^b^	NR	NR	NR	NR	Hospital ED	USA
Dai 2021 [37]	NR	NR	NR	NR	Psychiatric hospital	Taiwan
Tohira 2021 [41]	NR	51%	63 (range: 36–81)	NR	Ambulance	Australia
Chen 2022 [42]	779	28.1%	82.5 (SD 6.5)	Han Chinese (98.5%), Other (1.5%)	Hospital inpatients	China
Fu 2022 (a) [43]	300	48.3%	NR	NR	Multiple healthcare settings (inpatient, ED, outpatient)	USA
Fu 2022 (b) [44]	302	NR	NR	NR	NR	USA
Ge 2022 [45]	10 516	NR	NR	NR	NR	NR
Maclagan 2023 [46]	44 674	55.4%	74.7	NR	Primary care	Canada
Pagali 2023 [47]^c^	4351	NR	NR	NR	Multiple hospital sites	USA
St Sauver 2023 [48]^d^	400	52.2%	NR	White (90.8%), Black (1.6%), Asian (5.1%), other/mixed (2.5%)	Multiple healthcare providers (clinics)	USA

NR indicates that this information was not reported.

^a^If mean and standard deviation were not reported, alternative statistics listed where available.

^b^Note that this study refers to the same NLP tool as Patterson, 2019 [35]; however, it has been further refined in this study.

^c^Note that this study refers to the same NLP tool as Fu, 2022 (a) [43], and this is an external validation study.

^d^Note that this study refers to the same NLP tool as Fu, 2022 (a) [43]; however, it has been further refined in this study.

**Table 2:** Summary of methods used in each study

Study	Ageing syndrome studied	NLP methodology^a^	Source(s) of text	*n* of text notes in dataset	Testing/Validation method	Gold-standard/ comparator
Jarman 2010 [27]	Falls	Text classification—machine learning	Outpatient notes	5009	Train-test split	Manual text review
Toyabe 2012 [28]	Falls	Text classification—rule based	NR	2590	Train-test split	Manual text review
McCart 2013 [29]	Falls	Text classification—machine learning	Multiple document types	26 010	Train-test split & external validation	Recorded ICD-9 and e-codes in EHR
Reuben 2017 [30]	Dementia	Text classification—rule based	NR	NR	Tested on full dataset	Manual text review
Kharrazi 2018 [31]	Faecal incontinence, dementia, falls	Named entity recognition	Various sources, e.g. outpatient visit notes, home care notes, discharge summaries, emails	792 331^b^	A sample of notes used for testing	Manual text review
Chen 2019 (a) [32]	Faecal incontinence(i), dementia (ii), falls (iii), urinary incontinence(iv)	Text classification—machine learning	NR	NR	Train-test split	Manual text review
Chen 2019 (b) [33]	Faecal incontinence (i), dementia (ii), falls (iii), urinary incontinence(iv)	Text classification—deep learning	NR	8442	Train-test split	Manual text review
Gori 2019 [34]	Urinary incontinence (after radical prostatectomy)	Text classification—machine learning	Combination of progress notes, discharge summaries, telephone call notes and radiology reports	528 362	A sample of notes used for testing	Manual text review
Patterson 2019 [35]	Falls	Text classification—rule based	ED provider notes	1584	Train-test split	Manual text review
Topaz 2019 [36]	Falls	Text classification—machine learning	Various sources, e.g. admission notes, progress notes, physiotherapy notes	1 149 586	A sample of patients used for testing	Manual text review
Dolci 2020 [38]	Falls	Named entity recognition	Inpatient notes	NR	Train-test split & external validation	Manual text review
Moorthi 2020 [39]	Sarcopenia, frailty	Named entity recognition	Clinical notes	19 188	Sample of identified cases and controls selected for review	Manual text review
Patterson 2020 [40]	Falls	Text classification—rule based	NR	NR	Train-test split	Manual text review
Dai 2021 [37]	Dementia	Text classification—machine learning	Discharge summaries	500	Train-test split	Manual text review
Tohira 2021 [41]	Falls	Text classification—machine learning	Ambulance records	9447	Train-test split	Manual text review
Chen 2022 [42]	Delirium	Text classification—rule based	Physician medical notes, nursing notes	779	Tested on full dataset	Clinician diagnosis
Fu 2022 (a) [43]	Delirium	Text classification—rule based	Inpatient records (247), observation records (55), emergency records (186), outpatient records (127)	615	Train-test split	Manual text review
Fu 2022 (b) [44]	Falls	Text classification—deep learning	Clinical notes/documents	5447	Train-test split	Manual text review
Ge 2022 [45]	Delirium	Text classification—machine learning	Sentences from a variety of clinical notes	200 471^c^	Train-test split & external validation	Manual text review
Maclagan 2023 [46]	Dementia	Text classification—machine learning	Primary care notes	5 663 327	*K*-fold cross validation	Validated health administrative algorithm^d^
Pagali 2023 [47]	Delirium	Text classification—rule based	NR	NR	External validation study for Fu, 2022 (a)	Any one of three criteria^e^
St Sauver 2023 [48]	Delirium	Text classification—rule based	NR	NR	Train-test split	Manual text review

NR indicates that this information was not reported.

^a^If multiple NLP algorithms have been tested, the best performing algorithm is listed (based on F1-score, or alternative performance metrics if not available).

^b^Calculated using the mean number of notes per patient.

^c^Number of sentences.

^d^Algorithm identifies patients as having dementia if: patients with ≥1 hospitalisation and/or ≥3 physician visits for dementia within 2 years (30-day gap between each visit) and/or a prescription for a cholinesterase inhibitor.

^e^Three criteria were: clinician diagnosis of delirium or encephalopathy by ICD-10-CM codes, positive nursing DTS-bCAM assessment for delirium and detection of delirium diagnosis by the NLP-CAM algorithm.

The results of the QUADAS-2 quality assessments are listed in Appendix Table S3. There were eight studies that were categorised as having a high degree of concern in relation to their methods for patient selection, two studies that had a high degree of concern in relation to the index test, five studies that had a high degree of concern in relation to the reference standard and four studies that had a high degree of concern in relation to flow and timing. Only 8 of the 22 studies (36%) had a low degree of concern across all domains.

Performance metrics of the studies listed by ageing syndrome and NLP methodology are shown in Table 3 and Appendix Table S4, respectively. Predictive performance of the NLP algorithms varied across the studies (F1-score 0.57–0.95), with sensitivity (recall) ranging from 57.1% to 100%, and specificity ranging from 84.0% to 100%. Positive Predictive Value (precision) ranged from 47.0% to 100%, and Negative Predictive Value ranged from 93.5% to 100%, although this metric was only infrequently reported. Accuracy ranged from 93.2% to 97.1% and was also not frequently reported. The NLP algorithms did not show clear superior performance for any of the ageing syndromes or categories of methods.

**Table 3:** Performance metrics of NLP algorithms, listed by ageing syndrome

Study	Precision (PPV)	Recall (sensitivity)	F1-score	Specificity	NPV	Accuracy
Dementia						
Reuben, 2017 [30]	NR	63%	NR	NR	NR	NR
Kharrazi, 2018 [31]^a^	NR	87.5%–100%	NR	95.4%–100%	NR	NR
Chen, 2019 (a) [32]	62.5%	100%	0.769	NR	NR	NR
Chen, 2019 (b) [33]	71.4%	100%	0.863	NR	NR	NR
Dai, 2021 [37]	100%	58.3%	0.737	NR	NR	NR
Maclagan, 2023 [46]	85.2%	71.5%	0.772	99.8%	99.7%	NR
Sarcopenia and frailty						
Moorthi, 2020 [39]	93.3%	NR	NR	NR	NR	NR
Delirium						
Chen, 2022 [42]	NR	61.4%	NR	85.4%	NR	NR
Fu, 2022 (a) [43]	NR	91.9%	NR	100%	NR	96.7%
Ge, 2022 [45]^b^	NR	NR	NR	NR	NR	NR
Pagali, 2023 [47]	NR	80%	NR	NR	NR	NR
St Sauver, 2023 [48]	NR	64%	NR	84%	NR	NR
Falls						
Jarman, 2010 [27]	88.0%	81.1%	0.844	96.7%	NR	93.2%
Toyabe, 2012 [28]	81%	87%	0.840	98%	NR	NR
McCart, 2013 [29]	70.6%–86.0%	74.3%–86.0%	0.749–0.858	94.2%–96.9%	93.5%–96.9%	NR
Kharrazi, 2018 [31]^a^	NR	87.5–100%	NR	95.4–100%	NR	NR
Chen, 2019 (a) [32]	86.4%	82.6%	0.844	NR	NR	NR
Chen, 2019 (b) [33]	78.6%	95.7%	0.863	NR	NR	NR
Topaz, 2019 [36]	88.4% (86.5–89.0)	90.1% (88.1–91.3)	0.890 (0.879–0.900)	NR	NR	NR
Patterson, 2019 [35]	92% (86.2–95.5)	95.8% (90.5–98.6)	0.939	97.4% (95.2–98.7)	98.7% (96.9–99.4)	NR
Dolci, 2020 [38]^c^	47%	100%	NR	94%	100%	NR
Patterson, 2020 [40]	91.9% (86.1–95.5)	95.8% (90.5–98.6)	0.938	97.3% (95.2–98.7)	98.7% (96.9–99.4)	97.1% (95.0–98.3)
Tohira, 2021 [41]	88%	83.8%	0.859	NR	NR	NR
Fu, 2022 (b) [44]	93.9%	96.9%	0.954	92.9%	96.3%	NR
Incontinence						
Kharrazi, 2018 [31]^a^	NR	87.5%–100%	NR	95.4%–100%	NR	NR
Chen, 2019 (a) [32]	100%	66.7%	0.800	NR	NR	NR
Chen, 2019 (a) [32]	85.7%	85.7%	0.857	NR	NR	NR
Chen, 2019 (b) [33]	100%	66.7%	0.800	NR	NR	NR
Chen, 2019 (b) [33]	57.1%	57.1%	0.571	NR	NR	NR
Gori, 2019 [34]	NR	NR	0.860	NR	NR	NR

NR indicates that this information was not reported.

^a^NLP performance metrics in this study were not available for individual ageing syndromes, and refer to the performance of the NLP algorithm across all conditions included in the study.

^b^No performance metrics for patient-level classification. The presence of sentences indicating delirium was compared with the presence of ICD codes indicative of delirium, and a φ coefficient of 0.256 (95% CI: 0.252–0.259) was calculated indicating low association/agreement.

^c^Results from validation dataset.

Predictive performance of studies that used external validation as part of the testing strategy (*n* = 4) also varied (F1-score 0.749–0.858), with sensitivity (recall) ranging from 74.3% to 100% and specificity ranging from 94% to 96.9%. The positive predictive value (precision) ranged from 47.0% to 86.0%, and the negative predictive value ranged from 93.5% to 100%. Accuracy was not reported in any of the studies that performed external validation.

## Discussion

### Summary of the main results

This systematic review has found evidence that it is feasible to use NLP algorithms to identify ageing syndromes in EHRs and that it is possible to do so with acceptable diagnostic accuracy. A variety of methods were used across the different studies; however, these performed comparably well with no clearly superior method. The systematic review also demonstrated that although there has been some research on this topic, there has been a greater focus on some ageing syndromes such as falls, dementia and delirium. In contrast, there has been very little research on using NLP algorithms to identify other ageing syndromes such as sarcopenia that were only recently recognised as a disease according to the International Classification of Diseases (ICD) [49, 50] and none on multimorbidity/MLTCs that are not recognised in the ICD. Importantly, most studies to date have been performed in the USA, and it is unclear whether NLP algorithms conducted in different EHRs in different health care systems in other countries will have acceptable diagnostic accuracy.

The most popular NLP validation method was to use a train-test split [17] rather than validation on an external dataset. This method provides a good understanding of how the NLP algorithm performs on unseen documents from the same data source [51], but does not give any indication of how the NLP algorithm would perform on documents from other data sources (i.e. other hospitals or clinics). It is likely that different datasets (especially in different countries) use different language and style to record clinical notes, and such external validation is essential to ensure that NLP algorithms are robust to this variation in language and terminology [52]. A minority of studies included this step in the assessment of their NLP algorithms, and the results of these studies demonstrated acceptable performance, therefore indicating generalisability of these algorithms.

Most studies in this review used manual text review to establish a gold standard, for comparison against predictions from the NLP algorithms. This method is likely to provide a reliable indicator of the presence of some ageing syndromes such as falls or incontinence, the diagnostic features of which are likely to be clearly identifiable from clinical texts. However, manual text review is more difficult when identifying ageing syndromes that have more complex diagnostic criteria such as sarcopenia [53] and dementia [54], particularly where (as in the case of sarcopenia) the diagnosis relies on objective measurements that may not have been performed. Complex diagnostic criteria relying on objective measurements may similarly be challenging for the NLP algorithms, as extraction and interpretation of these measurements currently lies outside the scope of the algorithms in the current published literature. An additional consideration is that manual text review requires expert human annotators [55], and this task can be highly time consuming, especially when using large datasets that are preferable for optimising the training of NLP algorithms [56].

### Limitations of the evidence included in the review

The QUADAS-2 quality assessments of the studies indicated that whilst some of the included studies were well designed, some studies had limitations in their methods for patient selection, application of the index test, application of the reference standard and flow. For example, in relation to patient selection, some studies did not use a consecutive or random sample of patients and instead selected patients based on the presence of the ageing syndrome (e.g. indicated by a clinical code), sometimes with matched controls, and in one instance without a matched control group. These methods of patient selection have the potential to lead to bias by exaggerating the accuracy of the NLP algorithms [57]. With regards to the index test, one study [45] performed classifications to identify presence of delirium on a sentence level, rather than classifying overall delirium status on a patient level. This method is unlikely to be applicable to clinical practice, as it is not clear how it could be used by clinical staff to identify patients with delirium.

Some studies (for example, [39]) did not follow best practice with regards to the reference standard and flow. In this study, NLP predictions were made first and were then subsequently manually reviewed by two clinicians. During this manual review process, the clinicians were aware of how the text had been labelled by the NLP algorithm and thus could be biased by this awareness of the NLP results. Additionally in this study, a random sample of only 50 patients were selected from the negative control group (patients not identified with delirium by NLP) for manual text review, in contrast to manual text review for all positive cases. This study design is likely to lead to an overestimation of the diagnostic performance of the NLP algorithm [58]. Finally, there were some limitations in relation to the reporting of performance metrics of the NLP algorithms in many of the studies. Many studies did not report metrics such as sensitivity and specificity, and where metrics were reported, the source data (i.e. two-by-two tables) were often not reported.

### Limitations of the review process

Whilst we aimed to ensure that our search strategy was as broad as possible to capture all relevant studies, it remains possible that this search strategy may have missed some studies. We included both biomedical literature databases and computer science literature databases in order to limit this possibility. Given the heterogeneity of methods, metrics and populations, it was not possible to undertake meta-analysis or to formally test for publication bias. Our ability to interpret the results was constrained by the lack of a commonly accepted taxonomy for categorising types of NLP algorithms. We therefore created our own taxonomy that has been informed by the current literature [25, 26].

An additional limitation of this review relates to the age specified in the study inclusion criteria. We chose to include all studies that related to adult patients (>18 years old) to ensure that we did not inappropriately exclude any relevant studies. This of course may mean that some studies relate to younger adults where certain diagnoses (e.g. falls) may present differently and for different reasons. NLP algorithms from these studies may therefore not be generalisable to older patient groups. Additionally, not all studies reported patient demographics such as age, which was not something that we anticipated when designing the prespecified study protocol. However, among studies that did report age, mean age was at least 62.4 years old, suggesting that these studies were conducted in older population groups.

### Implications for clinical practice and research

This systematic review has demonstrated that it is possible to use NLP algorithms to identify patients with ageing syndromes in EHR and that these algorithms can identify ageing syndromes with acceptable accuracy. These NLP algorithms could therefore have valuable clinical utility, by identifying patients who have ageing syndromes and have either not been previously diagnosed or not correctly clinically coded in EHR. This would provide great benefit to patients as well as caregivers, by identifying individuals in need of specific care and support services relevant to their condition. In addition to these clinical benefits, these NLP algorithms can also provide value to clinical research by assisting in the identification and recruitment of patients with these ageing syndromes for research studies, where there are sometimes challenges in identifying study participants [59].

However, to ensure that these NLP algorithms are clinically useful, they need to be tested rigorously in a range of populations that reflect the patients for whom these NLP algorithms are intended to benefit. The balance of sensitivity versus specificity for a particular algorithm needs to be fine-tuned for the purpose of that algorithm; for clinical scenarios such as augmenting existing lists of coded diagnoses, specificity will be more important, as it will be important to avoid labelling people with an ageing syndrome that they do not have. As part of this testing, it is important that these NLP algorithms are evaluated against a reliable ‘ground truth’ diagnosis, which, in many cases, will be derived from expert review of text documents. Future studies should be designed and reported in line with current guidance for diagnostic accuracy studies, such as the STARD (Standards for Reporting of Diagnostic Accuracy Studies) guidelines [60].

## Supplementary Material

aa-23-2144-File002_afae135
