# Supplementary material for: The use of natural language processing for the identification of ageing syndromes including sarcopenia, frailty and falls in electronic healthcare records: a systematic review
Source: Age Ageing. 2024 Jul 6;53(7):afae135. doi: 10.1093/ageing/afae135 (PMC11227113; doi:10.1093/ageing/afae135)
Supplement: aa-23-2144-File002_afae135 [file aa-23-2144-file002_afae135.docx]

**The use of Natural Language Processing for the identification of ageing syndromes including sarcopenia, frailty and falls in Electronic Healthcare Records: A systematic review**

Appendices – Table of contents

[Appendix Table 1. Sample search strategy](#_Toc164181535)

Appendix Table 2. Further details on Natural Language Processing methodology and testing/validation strategy 7

Appendix Table 3. Quality assessment of included studies 9

Appendix Table 4. Performance metrics of NLP algorithms, listed by methodology 10

Appendix table 5 – Studies that were excluded from review, due to NLP predicting future events 12

Appendix material 1 – Data extraction sheet 14

### Appendix Table 1. Sample search strategy

| Database | Search strategy |
| --- | --- |
| PubMed | #1: Natural Language Processing  Keywords: “Natural Language Processing” OR “NLP” OR “Text mining” OR “Text data mining” OR “Sentiment Analys*” OR “Natural Language Processing”[MeSH] OR “Sentiment Analysis”[MeSH]  #2: Ageing syndromes  Keywords: “Ag*ing” OR “Ag*ing syndromes” OR “Dementia” OR “Delirium” OR “Frailty” OR “Sarcopenia” OR “Incontinence” OR “Falls” OR “Accidental Falls” OR “Multi-morbidity” OR “Multiple Long-term Conditions” OR (“Dementia”[MeSH]) OR (“Delirium”[MeSH]) OR (“Sarcopenia”[MeSH]) OR (“Urinary Incontinence”[MeSH]) OR (“Fecal Incontinence”[MeSH]) OR (“Accidental Falls”[MeSH])  #3: Healthcare Records  (medical AND record) OR “health care record*” OR “health record*” OR “Medical Records”[MeSH]  #1 AND #2 AND #3 |
| Embase | #1: Natural Language Processing  natural language processing$.mp. or natural language processing/ or nlp.mp. or text mining.mp. or text data mining.mp. or sentiment analys$.mp.  #2: Ageing syndromes  dementia/ or dementia.mp. or delirium/ or delirium.mp. or sarcopenia/ or sarcopenia.mp. or incontinence/ or incontinence.mp. or falling/ or fall$.mp. or accidental fall$.mp. or multiple chronic conditions/ or multi-morbidity.mp. or multiple long-term conditions.mp.  #3: Healthcare records  exp Electronic Health Records/ or healthcare record$.mp. or exp Medical Records Systems, Computerized/ or medical record$.mp. or exp Medical Records/    #1 AND #2 AND #3 |
| Medline | 1 exp Aging/ 161848  2 exp Frailty/ 5968  3 exp Dementia/ 187202  4 Delirium/ 10934  5 Confusion/ 4964  6 Sarcopenia/ 6948  7 exp Urinary Incontinence/ 34765  8 exp Fecal Incontinence/ 10477  9 Accidental Falls/ 27140  10 exp Multimorbidity/ 1951  11 dementia.mp. [mp=title, abstract, original title, name of substance word, subject heading word, floating sub-heading word, keyword heading word, organism supplementary concept word, protocol supplementary concept word, rare disease supplementary concept word, unique identifier, synonyms] 126974  12 delirium.mp. [mp=title, abstract, original title, name of substance word, subject heading word, floating sub-heading word, keyword heading word, organism supplementary concept word, protocol supplementary concept word, rare disease supplementary concept word, unique identifier, synonyms] 18470  13 frailty.mp. [mp=title, abstract, original title, name of substance word, subject heading word, floating sub-heading word, keyword heading word, organism supplementary concept word, protocol supplementary concept word, rare disease supplementary concept word, unique identifier, synonyms] 16750  14 incontinence.mp. [mp=title, abstract, original title, name of substance word, subject heading word, floating sub-heading word, keyword heading word, organism supplementary concept word, protocol supplementary concept word, rare disease supplementary concept word, unique identifier, synonyms] 58185  15 falls.mp. [mp=title, abstract, original title, name of substance word, subject heading word, floating sub-heading word, keyword heading word, organism supplementary concept word, protocol supplementary concept word, rare disease supplementary concept word, unique identifier, synonyms] 56117  16 multi morbidity.mp. [mp=title, abstract, original title, name of substance word, subject heading word, floating sub-heading word, keyword heading word, organism supplementary concept word, protocol supplementary concept word, rare disease supplementary concept word, unique identifier, synonyms] 640  17 multiple long term conditions.mp. [mp=title, abstract, original title, name of substance word, subject heading word, floating sub-heading word, keyword heading word, organism supplementary concept word, protocol supplementary concept word, rare disease supplementary concept word, unique identifier, synonyms] 49  18 1 or 2 or 3 or 4 or 5 or 6 or 7 or 8 or 9 or 10 or 11 or 12 or 13 or 14 or 15 or 16 or 17  525690  19 exp Natural Language Processing/ 5289  20 exp Data Mining/ 10253  21 sentiment analys#.mp. [mp=title, abstract, original title, name of substance word, subject heading word, floating sub-heading word, keyword heading word, organism supplementary concept word, protocol supplementary concept word, rare disease supplementary concept word, unique identifier, synonyms] 0  22 natural language processing.mp. [mp=title, abstract, original title, name of substance word, subject heading word, floating sub-heading word, keyword heading word, organism supplementary concept word, protocol supplementary concept word, rare disease supplementary concept word, unique identifier, synonyms] 6651  23 text mining.mp. [mp=title, abstract, original title, name of substance word, subject heading word, floating sub-heading word, keyword heading word, organism supplementary concept word, protocol supplementary concept word, rare disease supplementary concept word, unique identifier, synonyms] 2492  24 text data mining.mp. [mp=title, abstract, original title, name of substance word, subject heading word, floating sub-heading word, keyword heading word, organism supplementary concept word, protocol supplementary concept word, rare disease supplementary concept word, unique identifier, synonyms] 22  25 NLP.mp. [mp=title, abstract, original title, name of substance word, subject heading word, floating sub-heading word, keyword heading word, organism supplementary concept word, protocol supplementary concept word, rare disease supplementary concept word, unique identifier, synonyms] 2134  26 19 or 20 or 21 or 22 or 23 or 24 or 25 17468  27 18 and 26 |
| CINAHL | #1: Natural Language Processing  (MH "Natural Language Processing+") OR "natural language processing" OR “NLP” OR “text mining” OR “text data mining” OR “sentiment analys*”  #2: Ageing syndromes  (MH “Dementia+”) OR “Dementia” OR (MH “Delirium+”) OR “Delirium” OR (MH “Frailty Syndrome+”) OR “Frailty” OR (MH “Sarcopenia+”) OR “Sarcopenia” OR (MH “Incontinence+”) OR “Incontinence” OR (MH “Accidental Falls+”) OR “Accidental Fall*” OR “Fall*”  #1 AND #2 |
| ACM Digital Library | #1: AllField:(((text OR data) AND mining) OR "natural language processing" OR "NLP" OR "sentiment analysis")  #2: AllField:(Ageing OR aging OR "ageing syndromes" OR "aging syndromes" OR dementia OR delirium OR incontinence OR frailty OR falls OR "accidental fall")  #3: Keyword:((medical AND record) OR "health care record" OR "health record")  #1 AND #2 AND #3 |
| IEEE Xplore | #1: ("Full Text & Metadata":"Text mining" OR "Full Text & Metadata":"Natural language processing" OR "Full Text & Metadata":"NLP" OR "Full Text & Metadata":"Sentiment Analysis")  #2: ("Full Text & Metadata":"Dementia" OR "Full Text & Metadata":"Delirium" OR "Full Text & Metadata":"Frailty" OR "Full Text & Metadata":"Sarcopenia" OR "Full Text & Metadata":"Incontinence" OR "Full Text & Metadata":"Falls" OR "Full Text & Metadata":"Accidental Falls" OR "Full Text & Metadata":"Multi morbidity" OR "Full Text & Metadata":"Multiple long term conditions")  #3: ("Full Text & Metadata":"Medical Record" OR "Full Text & Metadata":"Health Record" OR "Full Text & Metadata":"Healthcare Record")  #1 AND #2 AND #3 |
| Scopus | #1: TITLE-ABS-KEY(((text OR data) AND mining) OR "natural language processing" OR "NLP" OR "sentiment analys*")  #2:  TITLE-ABS-KEY(Ag*ing OR “ag*ing syndromes” OR dementia OR delirium OR incontinence OR frailty OR falls OR “accidental fall”)  #3: TITLE-ABS-KEY((medical AND record) OR "health care record*" OR "health record*")  #1 AND #2 AND #3 |

### Appendix Table 2. Further details on Natural Language Processing methodology and testing/validation strategy

| *Study* | *Further detail on NLP methods* | *Further detail on testing/validation strategy* |
| --- | --- | --- |
| Jarman 2010 [27] | Text miner to extract features (NER and negation status). Features then used to train a logistic regression model. Global frequency/Inverse Document Frequency (GF/IDF) weighting scheme used. | 60% of data for training, 20% for validation, 20% for testing |
| Toyabe 2012 [28] | Text mining studio software | 2,590 notes for testing |
| McCart 2013 [29] | Weighted term-by-document matrix with dimensionality reduction for features, Support Vector Machine (SVM) for classification | 70% of data for training, 30% for testing |
| Reuben 2017 [30] | Presence of terms without negation | Tested on validation data-set of 989 patients |
| Kharrazi 2018 [31] | Pattern-based NLP algorithm | 185 notes for testing |
| Chen 2019 (a) [32] | Conditional Random Fields (CRF) model for classification | Notes from 85 patients for training, 50 patients for tuning, 50 patients for testing |
| Chen 2019 (b) [33] | Deep-neural network | Notes from 85 patients for training, 50 patients for tuning, 50 patients for testing |
| Gori 2019 [34] | Word2Vec(Neural word embedding) for feature extraction, logistic regression modelling for classification | 200 notes for testing |
| Patterson 2019 [35] | Rule-based algorithm | 1084 notes for training, 500 for testing |
| Topaz 2019 [36] | Word-embedding and machine learning | Notes from 750 patients used for testing |
| Dolci 2020 [38] | Extraction of fall related terms using SQL queries on oracle database. | Notes from 120 patients for training, 120 for testing, and a further 298 for validation |
| Moorthi 2020 [39] | NDepth software (Named Entity Recognition of terms indicating condition) | Validation of 10,288 identified cases and 50 identified controls |
| Patterson 2020 [40] | Rule-based NLP algorithm | Tested on validation data-set of 500 notes |
| Dai 2021 [37] | Deep learning text classification (Hierarchical Attention Network – Global Vectors for Word representation (HAN-GloVe)) | 80% of data for training, 20% for testing |
| Tohira 2021 [41] | TF-IDF for feature extraction, SVM for classification | 70% of data for training, 30% for testing |
| Chen 2022 [42] | Frequency of delirium related keywords determined for each text. If > 11.14 keywords then text classified as delirium (threshold determined using ROC AUC curves) | Tested on full data-set of 779 notes |
| Fu 2022 (a) [43] | MedTaggerIE (NLP pipeline with feature extraction and classification) | 150 (50%) of the patients’ records used for algorithm testing |
| Fu 2022 (b) [44] | Text classification using a hybrid model of BERT (Bidirectional Encoder Representations from Transformers) and post-hoc heuristic rules | 242 patients for training set, 60 patients for testing set |
| Ge 2022 [45] | Text-classification of labelled sentences using a transformer model | 200,471 sentences in training data-set. 60% used for model training, 20% for hyperparameter tuning, and 20% for testing |
| Maclagan 2023 [46] | Dementia related terms were extracted as features. Texts also vectorised using TF-IDF. LASSO supervised machine learning model used to classify texts. | 5-fold nested cross-validation, 70% training and 30% testing in each fold |
| Pagali 2023 [47] | Delirium and CAM (confusion assessment method) concepts are extracted from the text. The texts are then classified using a rule-based approach | External validation study across 16 different hospital sites |
| St Sauver 2023 [48] | Delirium and CAM (confusion assessment method) concepts are extracted from the text. The texts are then classified using a rule-based approach | 400 notes in validation data-set, 50% of which was used for algorithm refinement, and 50% for algorithm testing |

### Appendix Table 3. Quality assessment of included studies

|  | **Patient selection** | | **Index test** | | **Reference standard** | | **Flow and timing** |
| --- | --- | --- | --- | --- | --- | --- | --- |
| *Study* | *Risk of bias* | *Concerns regarding applicability* | *Risk of bias* | *Concerns regarding applicability* | *Risk of bias* | *Concerns regarding applicability* | *Risk of bias* |
| Jarman 2010 [27] | High | Low | Low | Low | Low | Low | Low |
| Toyabe 2012 [28] | Unclear | Low | Low | Low | Unclear | Low | Low |
| McCart 2013 [29] | High | High | Low | Low | Low | Low | Low |
| Reuben 2017 [30] | High | High | Low | Low | Low | Low | Unclear |
| Kharrazi 2018 [31] | Unclear | Low | Unclear | Unclear | High | High | Low |
| Chen 2019 (a) [32] | Low | Low | Unclear | Low | Low | Low | Low |
| Chen 2019 (b) [33] | Low | Low | Low | Low | Low | Low | Low |
| Gori 2019 [34] | High | High | Low | Low | Low | Low | Low |
| Patterson 2019 [35] | Low | Low | Low | Low | Low | Low | Low |
| Topaz 2019 [36] | Low | Low | Low | Low | Low | Low | Low |
| Dolci 2020 [38] | Unclear | Low | Low | Low | Low | Low | Low |
| Moorthi 2020 [39] | Unclear | High | High | High | High | High | High |
| Patterson 2020 [40] | Low | Low | Low | Low | Low | Low | Low |
| Dai 2021 [37] | Low | Low | Low | Low | Low | Low | Low |
| Tohira 2021 [41] | Low | Low | Low | Low | Low | Low | Low |
| Chen 2022 [42] | Low | Low | Low | Low | Low | Low | Low |
| Fu 2022 (a) [43] | High | Low | Low | Low | Low | Low | Low |
| Fu 2022 (b) [44] | Low | Low | Low | Low | Low | Low | Low |
| Ge 2022 [45] | High | Low | Low | High | High | High | Low |
| Maclagan 2023 [46] | Low | Low | Low | Low | High | Low | Low |
| Pagali 2023 [47] | Low | Low | Low | Low | High | Unclear | Low |
| St Sauver 2023 [48] | High | Low | Low | Low | Low | Low | High |

### Appendix Table 4. Performance metrics of NLP algorithms, listed by methodology

| Study | Precision (PPV^1^) | Recall (sensitivity) | F1-score | Specificity | NPV^2^ | Accuracy |
| --- | --- | --- | --- | --- | --- | --- |
| **Text classification – Machine learning** | | | | | | |
| Jarman 2010 [27] | 88.0% | 81.1% | 0.834 | 96.7% | NR | 93.2% |
| McCart 2013 [29] | 70.6 - 86% | 74.3 - 86% | 0.749 – 0.858 | 94.2 - 96.9% | 93.5 - 96.9% | NR |
| Chen 2019 (a) [32] - i | 100% | 66.7% | 0.800 | NR | NR | NR |
| Chen 2019 (a) [32]- ii | 62.5% | 100% | 0.769 | NR | NR | NR |
| Chen 2019 (a) [32] - iii | 86.4% | 82.6% | 0.844 | NR | NR | NR |
| Chen 2019 (a) [32] - iv | 85.7% | 85.7% | 0.857 | NR | NR | NR |
| Gori 2019 [34] | NR | NR | 0.860 | NR | NR | NR |
| Topaz 2019 [36] | 88.4% (86.5 - 89) | 90.1% (88.1 - 91.3) | 0.890 (0.879 – 0.900) | NR | NR | NR |
| Dai 2021 [27] | 100% | 58.3% | 0.737 | NR | NR | NR |
| Tohira 2021 [41] | 88% | 83.8% | 0.859 | NR | NR | NR |
| Fu 2022 (a) [43] | NR | 91.9% | NR | 100% | NR | 96.7% |
| Ge 2022 [45]^3^ | NR | NR | NR | NR | NR | NR |
| Maclagan 2023 [46] | 85.2% | 71.5% | 0.772 | 99.8% | 99.7% | NR |
| **Text classification – Deep learning** | | | | | | |
| Chen 2019 (b) [33] - i | 100% | 66.7% | 0.800 | NR | NR | NR |
| Chen 2019 (b) [33] - ii | 71.4% | 100% | 0.833 | NR | NR | NR |
| Chen 2019 (b) [33] - iii | 78.6% | 95.7% | 0.863 | NR | NR | NR |
| Chen 2019 (b) [33] - iv | 57.1% | 57.1% | 0.571 | NR | NR | NR |
| Fu 2022 (b) [44] | 93.9% | 96.9% | 0.954 | 92.9% | 96.3% | NR |
| **Text classification – Rule based** | | | | | | |
| Toyabe 2012 [28] | 81% | 87% | 0.840 | 98% | NR | NR |
| Reuben 2017 [30] | NR | 63% | NR | NR | NR | NR |
| Patterson 2019 [35] | 92% (86.2 - 95.5) | 95.8% (90.5 - 98.6) | 0.939 | 97.4% (95.2 - 98.7) | 98.7% (96.9 - 99.4) | NR |
| Patterson 2020 [40] | 91.9% (86.1 – 95.5) | 95.8% (90.5 – 98.6) | 0.938 | 97.3% (95.2 – 98.7) | 98.7% (96.9 – 99.4) | 97.1% (95.0 – 98.3) |
| Chen 2022 [42] | NR | 61.4% | NR | 85.4% | NR | NR |
| Pagali 2023 [47] | NR | 80% | NR | NR | NR | NR |
| St Sauver 2023 [48] | NR | 64% | NR | 84% | NR | NR |
| **Named Entity Recognition** | | | | | | |
| Kharrazi 2018 [31]^4^ | NR | 87.5-100% | NR | 95.4-100% | NR | NR |
| Dolci 2020 [38]^5^ | 47% | 100% | NR | 94% | 100% | NR |
| Moorthi 2020 [39] | 93.3% | NR | NR | NR | NR | NR |

NR: information not reported

^1^ Positive Predictive Value

^2^ Negative Predictive Value

^3^ No performance metrics for patient level classification. The presence of sentences indicating delirium was compared with the presence of ICD codes indicative of delirium, and a ϕ coefficient of 0.256 (95% CI: 0.252 – 0.259) was calculated indicating low association/agreement.

^4^ NLP performance metrics in this study were not available for individual ageing syndromes, and refer to the performance of the NLP algorithm across all conditions included in the study.

^5^ Results from validation data-set

### Appendix table 5 – Studies that were excluded from review, due to NLP predicting future events

| **Year** | **Title** | **Authors** |
| --- | --- | --- |
| 2023 | HomeADScreen: Developing Alzheimer's disease and related dementia risk identification model in home healthcare | Zolnoori et al. |
| 2020 | The Use of Data Mining Methods for the Prediction of Dementia: Evidence From the English Longitudinal Study of Aging | Yang et al. |
| 2019 | Importance of medical data preprocessing in predictive modeling and risk factor discovery for the frailty syndrome | Hassler et al. |
| 2012 | Mining geriatric assessment data for in-patient fall prediction models and high-risk subgroups | Marschollek et al. |

### Appendix material 1 – Data extraction sheet

**Data Extraction Sheet – NLP Systematic Review**

**Systematic review: The use of Natural Language Processing for the identification of ageing syndromes in Electronic healthcare records**

| Study title |  |
| --- | --- |
| Study author and date |  |
| Extracted by |  |
| Journal |  |
| Checked by |  |

| Location |  |
| --- | --- |
| Funding source |  |

| **Participants/Electronic Healthcare Record general description:**    **NLP algorithm Description:**    **Ageing syndrome:** | |
| --- | --- |
| Inclusion criteria | Exclusion criteria |
| . |  |

Population stats:

|  | **n =** |
| --- | --- |
| Sex of participants: |  |
| Mean age (S.D.): |  |
| Ethnic group: |  |
| Healthcare setting: |  |
| Source(s) of text (e.g. discharge summary, letter): |  |
| Number of notes: |  |

Methods:

- Programming language/software used to develop NLP algorithm?
- Method of NLP? (e.g. deep learning, text classification, NER etc.)
- Method of validation?

|  |
| --- |

Was the algorithm validated externally?

|  |
| --- |

Outcomes:

*Feasibility*

- Was it possible to apply the NLP algorithm on the EHR?
- Did the algorithm produce an output/result?
- Was the output interpretable?

|  |
| --- |

*Accuracy*

If multiple models are tested, give accuracy metrics for the best performing model:

| Sensitivity (Recall): |  |
| --- | --- |
| Specificity: |  |
| PPV (Precision): |  |
| NPV: |  |
| F score: |  |
| Micro F1 score: |  |
| Macro F1 score: |  |

Any additional accuracy metrics/results:

|  |
| --- |

Risk of Bias assessment:

Please see QUADAS-2 form and checklist

Other Findings:

Any additional findings/information:

|  |
| --- |
